# An interdisciplinary approach aiding the diagnosis of primary progressive aphasia: A case report

**DOI:** 10.1590/1980-57642016dn11-010014

**Published:** 2017

**Authors:** Nadia Shigaeff, Mayra Zanetti, Sibelle de Almeida Tierno, Ana Beatriz Galhardi Di Tommaso, Thais Cristina Marques, Fábio Gazelato de Mello Franco

**Affiliations:** 1 Hospital Israelita Albert Einstein, São Paulo, SP, Brazil, Universidade Federal do ABC, Santo André SP – Brazil.; 2 Hospital Israelita Albert Einstein São Paulo SP – Brazil.; 3 Universidade Federal de São Paulo, São Paulo SP – Brazil.

**Keywords:** aphasia, elderly, frontotemporal dementia, progressive primary aphasia

## Abstract

Frontotemporal dementia (FTD) is one of the most common causes of early-onset dementia with primary progressive aphasia (PPA) being the second-most-frequent form of this degenerative disease. Despite the similarity with progressive dementia (especially in early stages of Alzheimer´s disease), three types of PPA can be differentiated: semantic, agrammatic and logopenic (subtype discussed in this study). To date, no medications have been shown to improve or stabilize cognitive deficits in patients with PPA. We report the case of a 62-year-old woman with difficulty naming objects and planning. An interdisciplinary evaluation, including imaging and lab exams, together with neuropsychological and personality assessments, confirmed that the patient had logopenic PPA on the basis of repetition difficulty, phonemic and semantic paraphasias and absence of agrammatism. The timing of the assessment in this case, along with the resources available and commitment of an integrated interdisciplinary team, allowed a differential diagnosis (from other classical dementias) to be reached.

## Abstract (pt)

A demência frontotemporal (DFT) é uma das causas mais comuns de demências precoces e a Afasia Progressiva Primária (APP) é o segundo tipo mais prevalente desta patologia neurodegenerativa. Apesar das semelhanças entre as demências, especialmente considerando os estágios iniciais da demência de Alzheimer, é possível diferenciar três subtipos de APP: semântico, agramático/não-fluente e logopênico, que é o subtipo discutido no presente estudo. Até o momento não há estudos que tenham evidenciado melhora ou estabilização dos prejuízos cognitivos nos pacientes diagnosticados com APP por meio de controle medicamentoso. No presente estudo descreve-se o caso de uma idosa com 62 anos de idade que apresentou dificuldade para nomear objetos e fazer planejamentos. Após uma avaliação interdisciplinar, incluindo exames de neuroimagem e laboratoriais, associados a avaliações neuropsicológica e de personalidade, foi possível realizar o diagnóstico de APP subtipo logopênico, especialmente por causa da dificuldade de repetição, parafasias semântica e fonêmica e ausência de agramatismo. O momento da avaliação foi propício para realizar um diagnóstico diferencial de outras demências, contando também com os recursos disponíveis e o comprometimento de uma equipe interdisciplinar integrada.

## CASE REPORT

We report the case of a 62-year-old woman with seven years of education, whose daughter reported that her mother had a one-year history of difficulty naming objects and impaired planning. The patient had difficulty finding words and expressing herself appropriately. She had a 5-year history of diabetes and hypertension, both well controlled. The examination was unremarkable. Her global geriatric assessment showed no significant functional loss according to the account given by her daughter. There was no family history of similar problems. The final cognition screening showed worse results, particularly on language and executive function skills. The Mini-Mental State Examination score was 19, with deficits in temporal orientation, calculus and comprehension. The Brief Cognitive Battery showed impairment on strategies for spontaneous word recall, categorical verbal fluency, drawing clock face and naming objects.

Laboratory hematology and biochemistry tests were normal, except for mild eosinophilia. Magnetic Resonance Imaging (MRI) disclosed no remarkable findings.

In an interdisciplinary meeting, it was proposed that her condition could be a factitious disorder and she was referred for a personality assessment by a team of specialized psychologists.

At first, a regular neuropsychological assessment confirmed significant difficulties on the cognitive screening and also showed significant impairment in short-term immediate memory (visual), short-term working memory (visual and verbal) and long-term episodic memory (verbal). However, skills regarding visual memory capacity, praxis and speed in processing information were preserved. After adaptation for language difficulties, the results showed mild impairment in executive function, yet memory deficits were not observed. These mild executive function difficulties could be explained by the psychodynamic functioning of the elderly subject who showed signs of being quite anxious/depressed and insecure, requiring incentives and motivation to pursue the proposed tasks.

The Rorschach test (Comprehensive System) showed clear signs of a depressive process and problems controlling affective experiences. Her cognitive aspects were poor and problems in the affective sphere were likely to worsen her cognitive features. She showed a preference for emotion over thinking when solving problems, and thus problems in the affective area could lead to worse reaction and be more deleterious for her life.

The most notable aspect was the incongruity between the results of neuropsychological assessment and functional independence. A possible explanation is that the patient had a high level of education, which justifies a possible larger cognitive reserve. This may explain the independency of functionality in daily activities, especially those that she was used to performing. However, greater difficulty in learning and executing new behavioral patterns would be expected.

**Figure 1:**
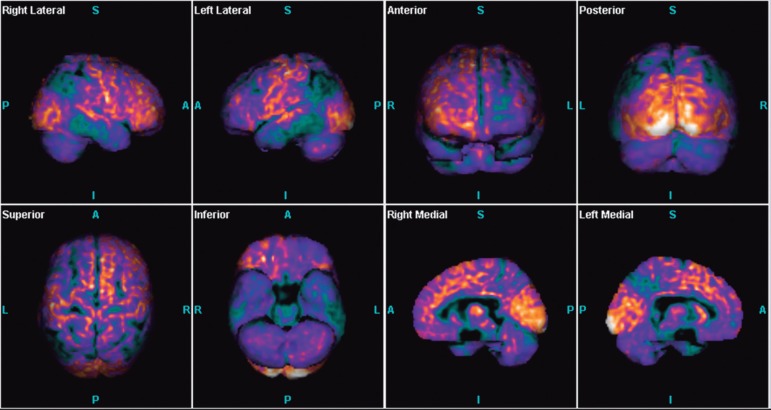
Positron Emission Tomography (PET) of the brain demonstrating hypometabolism in the parietal and temporal lobes extending to the frontal lobe, particularly in the left hemisphere.

As the result was negative for a factitious disorder, it was decided to expand the search with Positron Emission Tomography (PET) which revealed a major reduction in metabolism in the parietal and temporal lobes extending to the frontal lobe. These findings were much more pronounced in the left hemisphere (Figure 1).

A diagnosis of primary progressive aphasia (PPA) was reached based on clinical history, according to the criteria proposed by international expert consensus in 2011 (Table 1) together with the results of imaging studies and considering the early onset of symptoms given the young age of the patient.^1,2^

**Table 1:** Diagnostic classification criteria for primary progressive aphasia and its variants.

**A diagnosis of PPA requires all of the following features:**
• The most prominent clinical feature is difficulty with language.
• The language deficits are the principal cause of impaired activities of daily living.
• Aphasia is the most prominent deficit at symptom onset and for the initial phases of disease.
**In addition, the following four criteria** **must be answered negatively:**
• Pattern of deficits is better accounted for by other nonneurodegenerative nervous system or medical disorders.
• Cognitive disturbance is better accounted for by a psychiatric diagnosis.
• There are prominent initial episodic memory, visual memory, and visuoperceptual impairments.
• There is a prominent initial behavioral disturbance.

The logopenic variant was proposed as a diagnostic hypothesis and was based mainly on the aspects of slow speech rate and due to many hesitations and pauses in speech. In addition, she showed relative preservation of comprehension skills and grammar. Another aspect that contributed to a differential diagnosis were the difficulties objectively assessed by language tasks in the repetition of words or sentences.^3^

Regarding the cognitive profile in the PPA, the subject exhibited a possible logopenic variant of PPA, her complaints were still centered on language skills, but objectively impairment was also identified in the performance of some abilities of executive function.^4,5^

It is important to note that this case did not fulfill the criteria for diagnosis of behavioral variant frontotemporal dementia. One aspect that supports this notion is that the woman did not exhibit behavioral changes, as evidenced by the normal personality assessment results.^6^

There were no criteria indicating Alzheimer's diagnosis since language difficulties were very significant and there was no impairment in memory capacity. It is necessary to provide this explanation given that both these aspects can justify about 15% of errors in diagnosis because of the similarity of symptoms in the early course of Alzheimer and PPA diseases.^7^

Lastly, the diagnosis was explained to the patient and her daughter and they were guided on care strategies: it was suggested that the patient should start antidepressant treatment and cognitive rehabilitation, including speech therapy. Also, the patient´s daughter was invited to join a help group for relatives of patients with dementia, preparing her to cope with her mother´s cognitive impairment and the probable greater level of dependency in the future.
